# Prevalence Study of *Trichomonas gallinae* in Domestic Pigeons in Northeastern Beijing and Experimental Model of Trichomoniasis in White King Squabs Measuring In Situ Apoptosis and Immune Factors in Crop and Esophagus

**DOI:** 10.3390/ani14131869

**Published:** 2024-06-25

**Authors:** Aixin Ni, Yunlei Li, Adamu Mani Isa, Panlin Wang, Lei Shi, Jing Fan, Pingzhuang Ge, Linlin Jiang, Yanyan Sun, Hui Ma, Jilan Chen

**Affiliations:** State Key Laboratory of Animal Biotech Breeding, Institute of Animal Science, Chinese Academy of Agricultural Sciences, Beijing 100193, China

**Keywords:** *Trichomonas gallinae*, pigeon, infection, immune-related factors, apoptosis

## Abstract

**Simple Summary:**

Avian trichomonosis is a serious disease in pigeon production caused by *Trichomonas gallinae*. Previous research has described its morphology, epidemiology, diagnostic treatment and pathology, but there are only a few studies on the level of in situ apoptosis and immune factors in experimentally infected squabs. The prevalence of *Trichomonas gallinae* in five breeds ranged from 27.13% (White Carneau) to 43.14% (White King) in domestic pigeons in northeastern Beijing. By conducting an experimental model of trichomoniasis in White King squabs, measuring in situ apoptosis and immune factors in the crop and esophagus, we found that *Trichomonas gallinae* infection may lead to high mortality, trigger apoptosis and modulate immune-related factor expression in the crop and esophagus of pigeons.

**Abstract:**

*Trichomonas gallinae* (*T. gallinae*) is a flagellated protozoan and the causative agent of trichomoniasis, or canker, in birds. In the current study, the prevalence of *T. gallinae* was firstly investigated in five breeds. According to the results of the prevalence study, White King pigeons were selected as the experimental animals. A total of 135 White King squabs at one day of age were randomly divided into two groups and raised in separate isolators. The challenged group (N = 100) was challenged intranasally with 5 × 10^6^ parasites/mL of the *T. gallinae* strain, and the control group (N = 35) was intranasally administered medium of equivalent volume. At 1, 2, 3 and 5 days post infection (DPIs), the crops and esophagi were collected for RNA extraction and formaldehyde fixation. The results showed that prevalence of *T. gallinae* in the five breeds ranged from 27.13% (White Carneau) to 43.14% (White King). After the challenge, mild microscopic lesions were observed in both tissues. Apoptosis rates were higher in the challenged group than in the control group at 2 and 5 DPIs in the crop and at 1, 2 and 7 DPIs in the esophagus. For both tissues, relative expression of *IL-1β* increased dramatically at the beginning and decreased at 5 DPIs, and *TGF-β* increased stably in the challenged group.

## 1. Introduction

*Trichomonas gallinae* (*T. gallinae*) is a flagellated protozoan and the causative agent of trichomoniasis, or canker, in birds. It mostly appears as melon- or pear-shaped, with a size range of 7.0 μm~11.0 μm, and has an ovoid nucleus with a diameter of 2.5 μm~3.0 μm [1]. The lesions mainly impact the oropharynx, esophagus and crop. In severe cases of *T. gallinae* infection, the orbits, cervical soft tissues [2], liver and gizzard [3,4] may also be affected.

*T. gallinae* spreads rapidly and causes widespread implications. It has been found in many countries, such as Western and Southern Europe, the United Kingdom, Canada, Australia, Iran, Iraq and Mauritius [5,6,7,8]. In the United Kingdom, *T. gallinae* has caused mass death of greenfinches since 2006 [9]. Some researchers even believe that *T. gallinae* had spread to every corner of the world [10]. It is also a pathogen in a variety of birds, such as greenfinches, chaffinches, turtle pigeons, wood pigeons and band-tailed pigeons [11,12]. Domestic pigeons, feral pigeons and, less frequently, diurnal birds of prey, because of the predator–prey relationship, are susceptible to trichomoniasis [13]. However, severe lesions or clinical signs are primarily observed in squabs, rather than adult birds. The squabs receive the organism by infected “pigeon milk” from parents. Adult pigeons can be infected by the uptake of contaminated water or oral contact with affected pigeons [14]. It is believed that nearly all pigeons are carriers [2]. Pigeons infected by *T. gallinae* may have yellow plugs and exudate on the mucosa, but most of the infected pigeons do not show macroscopic lesions [14].

Many previous studies have focused on the morphology, epidemiology and pathology [3,4,5,6,7,8,9,13,15,16,17,18,19], but there are only a few on the level of in situ apoptosis and immune factors in experimentally infected squabs. Cell apoptosis is a manifestation of host self-protection and an important factor in regulating parasite-induced host immune responses [20]. Cytokines are the crucial mediator of the immune system when accounting for parasites [21]. Many studies have reported changes in apoptosis rate [22,23,24] and immune-related factors [25,26] after parasite infection. However, the levels of in situ apoptosis and immune factors were still not fully investigated after *T. gallinae* infections in experimentally infected squabs. Moreover, immune responses differ across infection stages [27].

In addition to measuring the in situ apoptosis and immune factors, the immune organ index (spleen index) was recorded. In birds, the spleen is a vital secondary lymphatic organ and the spleen index is known as the important indicator reflecting immune functions [28]. Therefore, in the current study, the prevalence of *T. gallinae* in domestic pigeons in northeastern Beijing was investigated, firstly, to determine the most suitable experimental breed for challenging. After challenging with *T. gallinae*, we further developed an experimental model of trichomoniasis and characterized the pathogenesis of *T. gallinae* infection by the evaluation of tissue lesions, apoptosis rates and the relative expression levels of immune-related factors in experimentally infected squabs.

## 2. Materials and Methods

### 2.1. Ethics Statement

All animal procedures were approved by the Animal Care and Use Committee of the Institute of Animal Science of the Chinese Academy of Agriculture Sciences (project number: IAS2019-67).

### 2.2. Prevalence of T. gallinae and Study Area

A total of 583 healthy pigeons from 5 pigeon breeds were used in the current study, including Tai Shen (116), Silver King (110), White King (102), Shen Wang (126) and White Carneau (129). Parasites were retrieved from the oral cavity by swab according to Mirzaei [29]. Briefly, a cotton swab was soaked in sterile saline. The excess fluid was removed and the swab was inserted into the oral cavity 3 times. Then, the swab sticks were pressed on a slide. Separate swab sticks, followed by separate slides, were used for each pigeon. After slide preparation, the sample-containing slides were examined under a light microscope for *T. gallinae* at 100× magnification. The protozoon was seen to move and was in the shape of a melon or pear. Samples were collected from a pigeon farm in the northeast of Beijing, China.

### 2.3. T. gallinae Challenge

The *T. gallinae* strain used in this study was described previously [30]. In brief, *T. gallinae* was isolated from the upper digestive tract of the natural infected White King pigeons. For these pigeons, the clinical signs were observed in the upper digestive tract, including caseous, proliferative, fibronecrotic lesions. The primary cultured *T. gallinae* strain was purified by differential centrifugation and density gradient centrifugation and then was identified by scanning electron microscopy and ITS1/5.8S/ITS2 region sequencing. For the challenging experiment, a total of 135 White King squabs at one day of age were randomly divided into two groups (the challenged group vs. the control group) and raised in two separate isolators with the same conditions. The challenged group (N = 100) was intranasally inoculated with 0.5 mL 5 × 10^6^ parasites/mL of the *T. gallinae* strain and repeated on 1, 2 and 3 days of age, while the control group (N = 35) was intranasally administered medium (without parasite) of equivalent volume. To avoid the possibility of infected parent pigeons transmitting *T. gallinae* to squabs, all experimental squabs were raised by artificial nursing. The squabs were fed with artificial synthetic pigeon milk (Tianyu hardcover pigeon milk, Tianyu, Bengbu, China) 12 times per day till 7 days of age and 6 times per day till 15 days of age. The experiment lasted for 15 days. At 1, 2, 3, 5 and 7 days post-infection (DPIs), three pigeons with similar body weight from each group were euthanized by cervical dislocation. The crops and esophagi were collected and divided into similar size. One piece was frozen in liquid nitrogen for further gene expression and the other was fixed in 10% neutral buffered formalin. Samples from 1, 2, 3 and 5 DPIs were used for gene expression, and samples from 7 DPIs were used for hematoxylin–eosin (H&E, Baton Rouge, LA, USA) histology. All samples were used for apoptosis analysis. The difference between tissue weight of the two groups was calculated by the average tissue weight in the control group minus the average tissue weight in the challenged group. The spleen index was calculated as the percentage of spleen weight in body weight.

### 2.4. T. gallinae Count

Mortality was recorded and dead pigeons were dissected to determine if they were infected with *T. gallinae* by microscope. At 1, 2, 3, 5 and 7 DPIs. Swab scraping samples were collected from the oral cavity of each pigeon in a 1.5 mL tube containing saline, centrifuged at 5000 rpm for 5 min. Supernatant was removed and precipitation was resuspended in 50 μL saline. Hemocytometers were used to count the numbers of *T. gallinae*.

### 2.5. Histopathological Study

Samples were fixed with 10% buffered formalin and cut into a small piece (1.0 cm length × 1.0 cm width). Samples were dehydrated in serial dilution of ethanol and embedded in paraffin wax. Finally, samples were cut into 5 μm thick sections manually with a microtome (Longshou, Shenyang, China), stained with hematoxylin and eosin (G1120, Solarbio, Beijing, China) for microscopic evaluation.

### 2.6. In Situ Detection of Apoptosis

An in situ cell death detection POD Kit (11684817910, Roche Molecular Biochemicals, Mannheim, Germany) was used for the TUNEL technique (terminal deoxynucleotidy1 transferase-mediated deoxyuridine triphosphate-biotin nick end labeling). Samples were incubated with 20 mg/mL proteinase K solution (1245680100, Merck, Darmstadt, Germany) for 30 min at 37 °C and then were washed with PBS 3 times for 5 min and incubated with TUNEL solution and mixed with 450 μL label solution and 50 μL of enzyme solution for 60 min at 37 °C in a moist and dark environment. The remaining 100 μL of label solution was used for the negative controls for alternate sections, which were then washed 3 times with PBS for 5 min and observed under a fluorescent microscope subsequently.

### 2.7. RT-PCR Detection of Immune-Related Factors Expression

Total RNA was isolated using Trizol reagent (15596026, Invitrogen, Carlsbad, CA, USA). The concentration and quality of RNA were evaluated using the NanoDrop 2000 (Thermo, Waltham, MA, USA). RNA was stored at −80 °C for further use. Total RNA was reverse transcribed into cDNA using PrimeScript RT Reagent Kit (RR047A, TaKaRa, Kusatsu, Japan) following the manufacturer’s instruction.

RT-PCR was performed using the ABI QuantStuio 7 Flex Real-time Detection System (Thermo, Waltham, MA, USA) and PrimeScript One Step RT-PCR Kit (RR055, TaKaRa, Shiga, Japan). Each 10 μL PCR mixture contained 5 μL of SYBR Premix Ex Taq™ II, 0.5 μL (10 pM) of each primer, 0.2 μL of ROX Reference Dye II (50×), 1.5 μL of cDNA (100 ng) and 2.3 μL of ddH2O. After an initial denaturing at 95 °C for 3 min, there were 40 cycles of amplification (95 °C for 30 s and 60 °C for 34 s), followed by thermal denaturing (95 °C for 15 s, 60 °C for 60 s and 95 °C for 15 s) to generate melting curves to verify amplification specificity. β-actin was amplified in the same plates as a house-keeping gene. Primers (Table 1) were designed using Premier 5.0 and their annealing specificity was confirmed using Oligo 6.0. Samples were assayed in triplicate for standard curves. The amplification efficiency of transcripts of interest and the internal standard (β-actin) was consistent. Dissociation curves verified that amplification was specific. The relative abundance of transcripts was calculated from 2^−ΔΔCT^.

### 2.8. Statistical Analysis

Data are presented as mean ± SD and analyzed using SAS 9.2 (SAS Institute Inc., Cary, NC, USA). Statistical analysis of the data was performed using one-sample *t* test. Chi-square was used to evaluate the difference of cumulative mortality between the two groups and *p* ≤ 0.05 was considered as significant.

## 3. Results

### 3.1. Prevalence of T. gallinae

The prevalence of *T. gallinae* in northeastern Beijing is shown in Table 2. For each breed, the prevalence of adult pigeons was 22.58%, 10.00%, 37.14%, 26.67% and 8.33% in Tai Shen, Silver King, White King, Shen Wang and White Carneau, respectively. The prevalence of squabs was 57.41%, 58.00%, 56.25%, 37.88% and 43.48% in Tai Shen, Silver King, White King, Shen Wang and White Carneau, respectively. The prevalence of *T. gallinae* in northeastern Beijing was 34.31% overall. The prevalence of White King pigeon was 43.14%, the highest among the five breeds (*p* = 0.03). Average infection rate of males was 26.92%, significantly higher than females (16.03%, *p* = 0.01). Adult pigeons were more resistant than squabs, as the infection rates were 21.47% and 49.08%, respectively (*p* ≤ 0.0001).

### 3.2. Cumulative Mortality and Body Weight Changes after Challenging with T. gallinae

**Figure 1:**
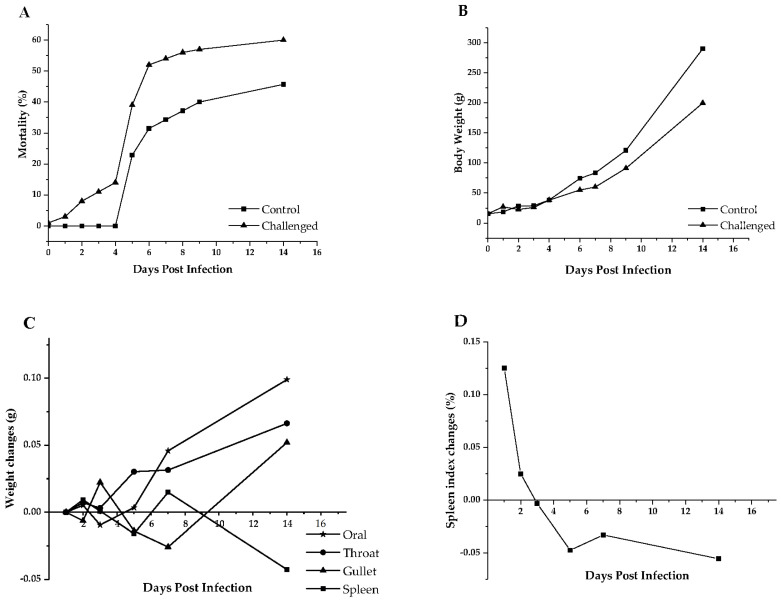
Effect of *T. gallinae* infections on mortality, body weight and digestive tract tissue weight and spleen index. (**A**,**B**) show changes in mortality and body weight after infection with *T. gallinae*; (**C**) shows the weight changes of the control group minus the challenged group; (**D**) shows the spleen index changes of the control group minus the challenged group.

Microscopic examination of the oral cavity showed that number of *T. gallinae* increased gradually with DPIs in the challenged group and was absent in the control group (Table 3). Cumulative mortality between 1 DPIs and 14 DPIs is presented in Figure 1A. Values in the challenged group were higher than those in the control group at 4 DPIs and 6 DPIs (*p* ≤ 0.05). 

No differences in body weight were observed between the challenged and the control groups (Figure 1B). All tissues (except spleen) in the control group were heavier than those in the challenged group at 14 DPIs (Figure 1C). Spleen index changes in the control group were lower than the challenged group after 2 DPIs (Figure 1D). 

### 3.3. Pathological Changes in the Crop and Esophagus

**Figure 2:**
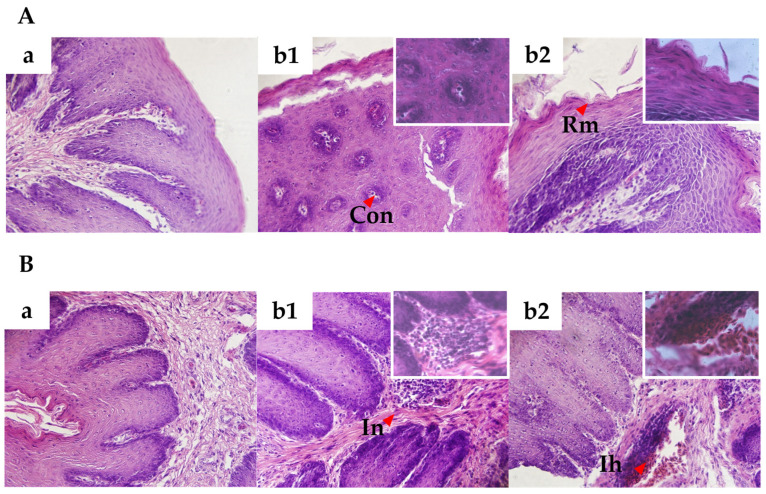
Histopathological observations in the crop and esophagus after the challenge (400× in the main figures and 1000× in the inserted figures). (**A**) Representative pathological images of *T. gallinae* infected crop at 7 DPIs. (**a**): the control group; (**b1** and **b2**): the challenged group, Con: congestion, Rm: mild rough mucosa. (**B**) Representative pathological images of *T. gallinae* infected esophagus at 7 DPIs. (**a**): the control group; (**b1** and **b2**): the challenged group, In: increased number of inflammatory nodules, Ih: mild infiltration of heterophils.

Hematoxylin and eosin staining were used to study the pathological changes in the crop and esophagus tissues at 7 DPIs (Figure 2). There were no histopathological injuries of individuals in the control group. Mild microscopic lesions were obvious in the crop and esophagus of the challenged group, including congestion, thickened mucosal layer, mild rough mucosa, increased number of inflammatory nodules and mild infiltration of heterophils. 

### 3.4. Assessment of Apoptosis

**Figure 3:**
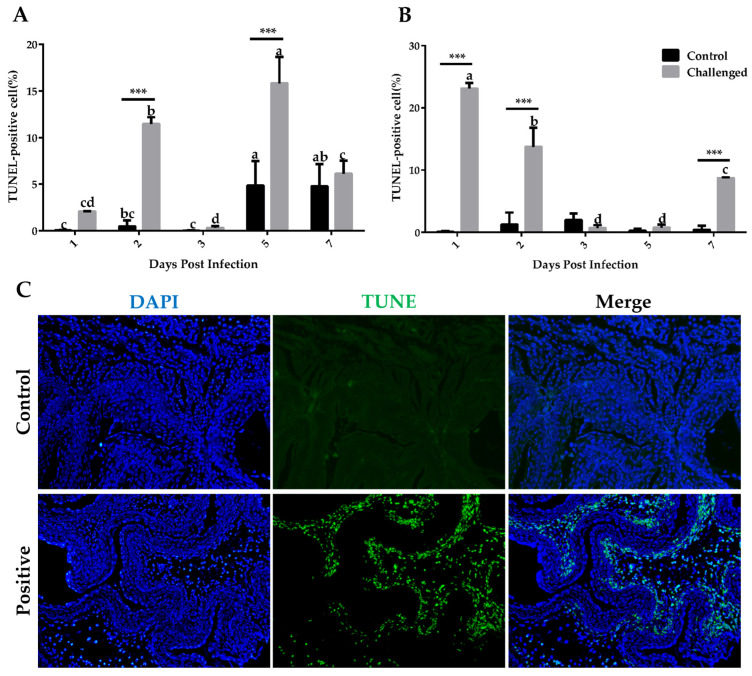
In situ apoptosis of the crop and esophagus tissues detected by the TUNEL method. (**A**) Quantification of the control and the challenged group at different days in the crop tissue that have acquired DNA strand breaks as observed in the TUNEL assay. SDs of three independent replicates are shown as error bars. (**B**) Quantification of the control and the challenged group at different days in the esophagus tissue. *** Significant difference between two groups (*p* ≤ 0.001); non-matching letters within a treatment differ significantly at *p* ≤ 0.05. (**C**) Fluorescence (DAPI in blue and TUNEL in green) microscopy images of different groups. The DAPI + TUNEL (Merge) images are shown on the far-right panels.

Apoptotic cells in the crop and esophagus induced by *T. gallinae* were confirmed by TUNEL assay (Figure 3A,B). In the challenged group, the fragmentation of chromosomal DNA congregated into small, condensed bodies, while the control group had intact chromosomal DNA and was not TUNEL labeled (Figure 3C). Quantification of cells with positive TUNEL labeling revealed that the apoptosis rate was significant at 2 DPIs and 5 DPIs in the crop and at 1, 2 and 7 DPIs in the esophagus in comparison with the control group. In the challenged group, apoptosis rate in the crop at 5 DPIs and in the esophagus at 1 DPIs was significantly higher than other detected ages. 

### 3.5. Expression of Immune-Related Factors

**Figure 4:**
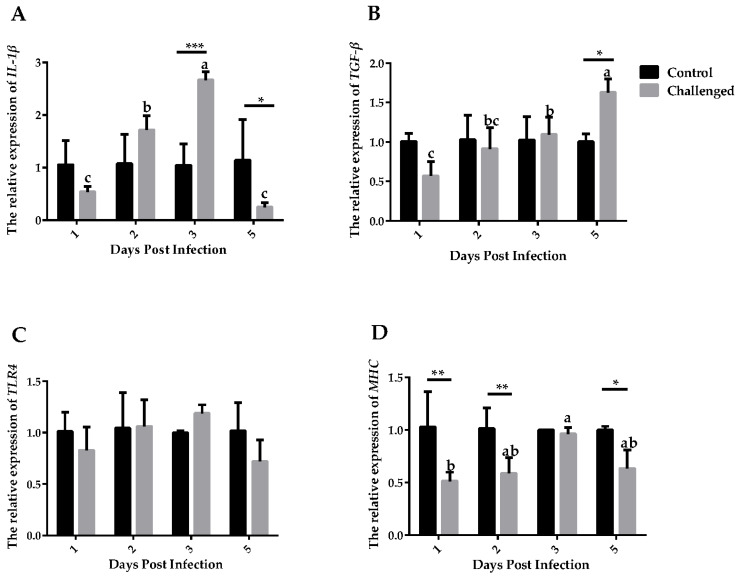
Relative mRNA expression of immune-related factors in the crop between the control group and the challenged group after the challenge. (**A**) Relative mRNA expression of *IL-1β*. (**B**) Relative mRNA expression of *TGF-β*. (**C**) Relative mRNA expression of *TLR4*. (**D**) Relative mRNA expression of *MHCII*. The relative expression was normalized with the control at the same time point. Significant difference between the control group and the challenged group is indicated as follows: * *p* ≤ 0.05, ** *p* ≤ 0.01, *** *p* ≤ 0.001; non-matching letters within a treatment differ significantly at *p* ≤ 0.05.

**Figure 5:**
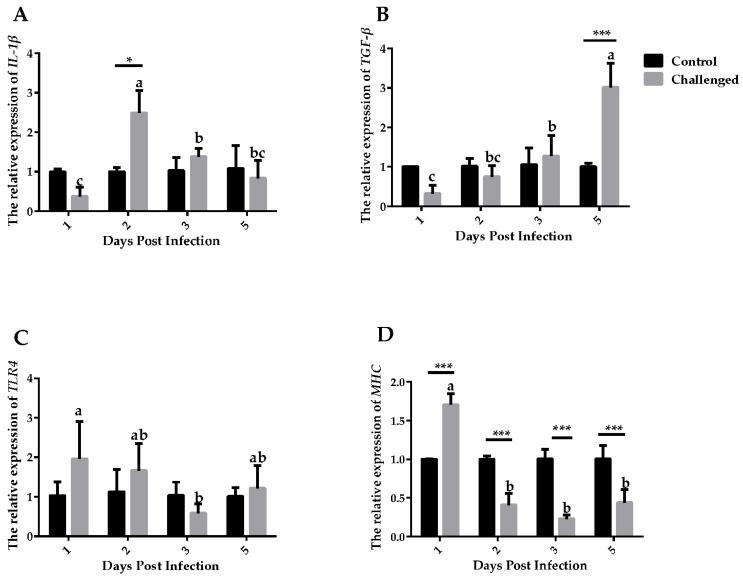
Relative mRNA expression of immune-related factors in the esophagus between the control group and the challenged group after the challenge. (**A**) Relative mRNA expression of *IL-1β*. (**B**) Relative mRNA expression of *TGF-β*. (**C**) Relative mRNA expression of *TLR4*. (**D**) Relative mRNA expression of *MHCII*. Significant difference between the control group and the challenged group is indicated as follows: * *p* ≤ 0.05, *** *p* ≤ 0.001; non-matching letters within a treatment differ significantly at *p* ≤ 0.05.

Expressions of *IL-1β*, *TGF-β*, *MHCII* and *TLR4* in the crop (Figure 4) and esophagus (Figure 5) were detected. *IL-1β* expressed significantly higher in the challenged group than the control group at 3 DPIs in the crop (*p* ≤ 0.001) and at 2 DPIs in the esophagus (*p* ≤ 0.05). *TGF-β* expressed significantly higher in the control group than in the challenged group at 5 DPIs in both tissues. Expressions of *TLR4* showed no significant differences between the challenged and the control groups at different days and tissues (*p* > 0.05). Expressions of *MHCII* were lower at 1, 2 and 5 DPIs in the challenged group than in the control group in the crop (*p* ≤ 0.05). Expressions of *MHCII* were higher at 1 DPIs, then statistically lower at 2, 3, and 5 DPIs in the esophagus (*p* ≤ 0.001). 

## 4. Discussion

The overall prevalence of *T. gallinae* in the study area was 34.31%, which is consistent with studies conducted by Feng et al. [31]. We found the prevalence between breeds, genders and ages showed significant difference. Salem also found significative differences between adults and squabs for *T. gallinae* [32]. It is necessary to explore the pathogenesis, apoptosis and immune-related factors and, thus, gain insight into a new approach for prevention.

In the current study, a trend was observed that body weight in the control group was higher than the challenged group after 4 DPIs. Consistent with this finding, many other researchers reported a decrease in body weight after parasitic infection [33,34]. However, no significant difference was observed for body weight between the two groups, which could be caused by the limited number of experimental animals. The mortality of squabs was significant from 4 DPIs to 6 DPIs, in agreement with Mesa’s results [35]. The high mortality of the control group in our study can be explained not only by the lack of maternally derived antibody in artificial synthetic pigeon milk, which was confirmed to be important in viability of offspring [36], but also by the process of artificial nursing. The current study confirmed that infection of *T. gallinae* could increase mortality.

Number of *T. gallinae* increased with days post infection in the challenged group. However, no clinical signs were observed in the infected squabs, which may be resulted from the low virulence of the infecting strain and the limited challenging time and dose. Histological examination of tissues showed congestion and thickened mucosal layer. Narcisi et al. [15] also reported a virulent strain of *T. gallinae* which could lead to the vascular congestion with perivascular cuffing and fatty degeneration of the hepatocytes in the liver, caseous masses in intestinal and gizzard surfaces, substernal membranes and pericardium. Infiltration of heterophils was observed from histological examination in the challenged group. In the previous studies, Brunthaler et al., Abaas et al., and Borji et al. also found the infiltration of heterophils in the infected birds [4,13,19]. Mild infiltration of heterophils was observed in the challenged group in the current study, suggesting the immune response of the pigeons to *T. gallinae* infections. 

Apoptosis, a form of programmed cell death, can be induced by parasitic infections, with the outcomes varying based on the specific host–parasite interaction [37]. In the current study, assessment of apoptosis by TUNEL revealed that *T. gallinae* infection could increase the apoptosis rate in the infected squabs. Li et al. [38] also reported the enrichment of apoptosis after *T. gallinae* infection in the KEGG enrichment analysis.

Eukaryotic parasites are difficult to clean up from the host once infected. The antigen component of the parasite is complicated and mutative. Parasites also have a complex life history, expressing specific antigens at specific stages [39]. Immune-related factors play a key role as mediators in the immune response to parasitic infection [40]. In the current study, we measured the expression of *IL-1β*, *TGF-β*, *MHCII* and *TLR4* by RT-PCR upon *T. gallinae* infection. 

*IL-1β* is a proinflammatory cytokine that is synthesized by inflammation and secreted by a variety of immune cells; thereby, it is essential for the host’s response to infection and injury [41]. In this study, *IL-1β* exhibited an abrupt rising trend at 3 DPIs in the crop and 2 DPIs in the esophagus, which may increase the expression of adhesion factors on endothelial cells, thus enabling transmigration of immunocompetent cells to sites of invasion [42]. It can be speculated that *IL-1β* played an immune-promoting role in the early stage of *T. gallinae* infection. *IL-1β* are produced by immune cells in a variety of different infections in animals or humans, primarily induced by LPS [43]. *TLR4* is the primary recognition molecule for inflammatory responses initiated by bacterial LPS [44]. Thus, no significant difference in *TLR4* expression between the challenged and the control groups may suggest that members of *TLR* family other than *TLR4* activated the activation of dendritic cells.

*TGF-β* is an immunosuppressive factor and its relative expression increases with the days post infection in both tissues. *TGF-β* plays an important role in a variety of parasitic infections and can regulate the level of neuronal inflammation induced by glial cells in *Plasmodium* infection [26]. *Heligmosomoides polygyrus* could also secrete *TGF-β* during the invasion phase to induce the production of regulatory T cells in mice [25]. However, considering that *TGF-β* is a cellular growth factor and both tissues exhibited cellular apoptosis, the increased level would be explained by the process of tissue repair in the apoptotic cells. Therefore, it can be proved that the expression of *TGF-β* increases after *T. gallinae* infection and suggested that *TGF-β* expressed from low to high may help the host to recover. *MHCII* are constitutively expressed in professional, immune antigen-presenting cells [45], interact mainly with immune cells, such as the T helper cells, and the peptide presented regulates how T cells respond to an infection [46]. Lower *MHCII* expression level in the challenged group indicated that phenotypic maturation of dendritic cells is inhibited [47]. Taken together, we confirmed the increased expression of immune-related factors against *T. gallinae* in the early stage of infection.

## 5. Conclusions

In general, the prevalence of *T. gallinae* in the domestic pigeons in northeastern Beijing is 21.47% for adults and 49.08% for squabs. In our study, squabs were infected by *T. gallinae* successfully under experimental conditions. The infection may lead to high mortality and triggers apoptosis and modulates immune-related factors expression in the crop and in the esophagus of pigeons.

## Figures and Tables

**Table 1:** Primer sequences and product size for immune-related factors measured in crop and esophagus in White King squabs challenged by *T. gallinae*.

Gene *	Accession Number	Primer Sequence (5′ to 3′)	Product Size (bp)
*IL* *-1β*	NM_001282824.1	F: TGGCGTTTGTCCCTGATTTG	56
		R: CGTCTCTTCATTCAGGCTGC	
*TGF-β*	XM_005502350.2	F: ACTGAGACTGTGCGTGAGTG	104
		R: AAGATGTCTCCGTTGGGCTG	
*MHCⅡ*	XM_013371611.1	F: GGAACCATCGTGCCACCC	98
		R: GGCCAGAACTTGTCCACGTA	
*TLR4*	XM_005498384.2	F: ACGTGCATGGGACTGAATGT	134
		R: GTATGGAGCTGGCACCTTGT	
*β-actin*	NM_001199586.1	F: GAGAAATTGTGCGTGACATCA	152
		R: CCTGAACCTCTCATTGCCA	

* *IL-1β* = interleukin 1 beta, *TGF-β* = transforming growth factor beta, *MHCⅡ* = HLA class II histocompatibility antigen, *TLR4* = toll-like receptor 4, *β-actin* = actin beta.

**Table 2:** Prevalence of *T. gallinae* in five breeds in northeastern Beijing.

Breed	Male	Female	Adults	Squabs	Average
Tai Shen	29.03% (31) *	16.13% (31)	22.58% (62)	57.41% (54)	38.79% (116)
Silver King	16.67% (30)	3.33% (30)	10.00% (60)	58.00% (50)	31.82% (110)
White King	45.71% (35)	28.57% (35)	37.14% (70)	56.25% (32)	43.14% (102)
Shen Wang	30.00% (30)	23.33% (30)	26.67% (60)	37.88% (66)	32.54% (126)
White Carneau	10.00% (30)	6.67% (30)	8.33% (60)	43.48% (69)	27.13% (129)
Average	26.92% (156)	16.03% (156)	21.47% (312)	49.08% (271)	34.31% (583)

* Numbers of detected pigeons are presented in parentheses.

**Table 3:** *T. gallinae* numbers in the oral cavity in White King squabs challenged with *T. gallinae*.

Days Post Infection	1	2	3	5	7
Control group	0	0	0	0	0
Challenged group (parasite/mL)	1.52 × 10^4^	1.68 × 10^4^	3.25 × 10^4^	7.22 × 10^4^	3.16 × 10^6^

## Data Availability

The datasets used during the current study are available from the corresponding author upon reasonable request.
